# Chimeric Antigen Receptor-Engineered Natural Killer (CAR-NK) Cell Therapy in Acute Myeloid Leukemia: A Systematic Review of the Literature

**DOI:** 10.7759/cureus.94807

**Published:** 2025-10-17

**Authors:** Mayra Steffania Novoa Arias

**Affiliations:** 1 General Medicine, Virrey Solis, Bogota, COL

**Keywords:** acute myeloid leukemia, car, chimeric antigen receptor, immunotherapy, natural killer cells

## Abstract

Chimeric antigen receptor (CAR)-engineered natural killer (NK) cells are attracting considerable interest as a potential therapeutic option in acute myeloid leukemia (AML), driven by the search for safe, scalable, and effective alternatives. To provide an overview of the current state of the field, a systematic review was performed to evaluate the safety and efficacy of CAR-NK cells for AML. The findings suggest possible directions for future development and also highlight important obstacles that must be addressed before they can be used in clinical settings. Therefore, although CAR-NK therapy remains in its early stages, its eventual application in AML is possible if these barriers can be resolved.

## Introduction and background

Acute myeloid leukemia (AML) is a malignant disease that affects hematopoietic stem cells, characterized by uncontrolled proliferation and impaired differentiation of myeloid progenitors [1]. Globally, its incidence and mortality have almost doubled in the past three decades, rising from 63,840 to 119,570 cases between 1990 and 2017 and from 51,770 to 99,900 deaths [2]. These numbers underscore the aggressive course of AML and the biological diversity that makes predicting outcomes and guiding treatment so challenging.

Although most cases appear de novo, AML is influenced by chromosomal rearrangements and acquired mutations [1]. The classical “two-hit hypothesis” proposed by Gilliland [3] distinguishes class I mutations (FLT3, RAS, KIT), which activate proliferative pathways, from class II mutations (NPM1, CEBPA, AML1, PML/RARα), which affect cellular differentiation. Particularly, FLT3-ITD occurs in 30% of patients with a normal karyotype, and NPM1 mutations in approximately 60% [4,5].

Despite advances in molecular and genomic characterization that have driven the development of novel therapies, standard treatment for AML still relies on intensive chemotherapy and/or hematopoietic stem cell transplantation [6]; outcomes, however, remain poor, and five-year survival is particularly low among patients with adverse cytogenetics or those over 60 years of age, with overall survival ranging between 5 and 10% [7,8].

Therefore, the search for more effective therapies has brought attention to chimeric antigen receptor (CAR) T-cell therapy, motivated by its progress in treating refractory hematologic cancers [9,10]. Yet, the application of CAR-T therapy in AML faces multiple limitations, including severe adverse events such as cytokine release syndrome (CRS), negative immunomodulation by the tumor microenvironment, significant immunosuppression associated with pancytopenia, as well as high costs and extensive production times [11-13]. As a result, no CAR-T product has been approved for the treatment of AML.

In this field, CAR-engineered natural killer (CAR-NK) cells are gaining attention. NK cells are members of the innate lymphoid group, and integrate activating and inhibitory receptors such as KIRs, C-type lectins, and natural cytotoxicity molecules to distinguish healthy from malignant cells. Specifically, they recognize human leukocyte antigen (HLA) class I molecules, respond to the absence of MHC-I through the “missing-self” mechanism, detect stress-induced ligands, and mediate tumor lysis via death receptors, antibody-dependent mechanisms, and effector proteins like perforin and granzymes [14,15]. These processes help NK cells to exert powerful antitumor responses while maintaining tolerance to healthy tissues. 

The advance from early constructs with CD3ζ signaling to fourth-generation platforms that integrate costimulatory domains (CD28, 4-1BB), cytokine transgenes such as IL-15, safety switches like iCasp9, and more sophisticated regulatory elements has broadened CAR-NKs' applicability, improving their cytotoxicity, expansion, and scalable production [16].

In parallel, allogeneic sources expand the therapeutic landscape, and the most exciting frontier lies in induced pluripotent stem cells (iPSC)-derived NK cells, which are renewable, genetically malleable, and primed for standardized CAR design, they hold promise to overcome many of the bottlenecks that have affected earlier platforms (peripheral blood NKs, NK-92 lines, cord blood cells) such as heterogeneity, limited expansion, loss of persistence for irradiation, and immature phenotypes [16,17].

Encouraging preclinical and early clinical studies in B-cell malignancies, multiple myeloma, and aggressive T-cell leukemias have demonstrated that CAR-NK therapy can provide meaningful antitumor activity with a consistently favorable safety profile related to other adoptive immunotherapies [18,19]. Therefore, the low rate of toxicity, antigen specificity, and feasibility for large-scale manufacturing of CAR-NK cells make them a potentially safer and more accessible therapeutic option for AML [18-20].

Against this backdrop, the present systematic review synthesizes the available clinical and preclinical evidence on CAR-NK therapy in AML, with the aim of providing a basis for future research and facilitating the development of more effective therapeutic strategies.

Methodology

The literature was searched from PubMed and Scopus by taking “AML”, “immunotherapy”, “natural killer cells”, and “chimeric antigen receptor” as keywords. The words were combined with “or” and “and” for joint search. It should search for clinical studies without date restrictions, limited to open-access articles in English or Spanish (Appendix). The last search was performed on April 5, 2024, following the process indicated by the Preferred Reporting Items for Systematic Reviews and Meta-Analyses (PRISMA) guidelines [21].

Eligibility Criteria

Eligible studies included clinical trials of any age or sex, and in vivo preclinical studies using human leukemic cells that evaluated CAR-NK therapy in AML. Studies were required to include a control group of any type and report at least two of the following outcomes: leukemic burden reduction, overall survival, tissue infiltration in bone marrow or spleen, and/or CAR-NK cell persistence. Randomized, quasi-randomized, and non-randomized designs were accepted, provided the full text was available in English or Spanish. Exclusion criteria included in vitro or ex vivo studies, investigations of other cell therapies (e.g., CAR-T, BiKE, TriKE), and studies using alternative methodologies.

Selection Process

The articles retrieved from the databases were organized by publication date. In the first stage, duplicates were removed by matching the year of publication, title, and authors using the Rayyan artificial intelligence platform [22]. The remaining articles were then screened by title and abstract; those that met the predefined eligibility criteria underwent full-text review to assess their relevance in greater detail. All stages of the selection process were carried out by one reviewer.

Data Collection Process

Data from eligible studies were extracted and analyzed using a custom template in Microsoft Excel (v16.0, Redmond, WA, USA). References were managed and documents organized with Zotero (v6.0.36). All stages of screening, extraction, and analysis were performed by a single reviewer.

Risk of Bias Assessment

Risk of bias in preclinical studies was assessed with the Systematic Review Centre for Laboratory Animal Experimentation (SYRCLE) tool [23], which covers 10 domains across six bias categories (selection, performance, detection, attrition, reporting, and other); judgments were recorded as Yes (Y) for low risk, No (N) for high risk, and Unclear (U) when information was insufficient. For clinical studies, methodological quality was appraised using the Joanna Briggs Institute (JBI) tool for quasi-experimental designs [24], comprising nine items spanning internal validity and statistical conclusion validity; a total score above 5 (five) was interpreted as good quality (i.e., lower risk of bias).

Synthesis Methods

Data synthesis was performed in Microsoft Excel (v16.0). For each study, leukemic burden, overall survival, tissue infiltration, and CAR-NK cell persistence were extracted into structured tables (ordered by first author), noting xenograft model, treatment/control cell types, point estimates, and the reported statistical tests with p-values. When numerical values were not provided, data were digitized and estimated from figures using WebPlotDigitizer (v5.2; Automeris, Dublin, CA, USA) [25]. Outcomes were summarized as mean ± SD; if SD could not be obtained, results were presented as median (range); and, when only SEM was reported, SD was derived from the sample size. Log-scaled figures were analyzed on the log scale and back-transformed. A meta-analysis was not performed due to heterogeneity and incomplete reporting of statistical measures in several studies.

## Review

**Figure 1:**
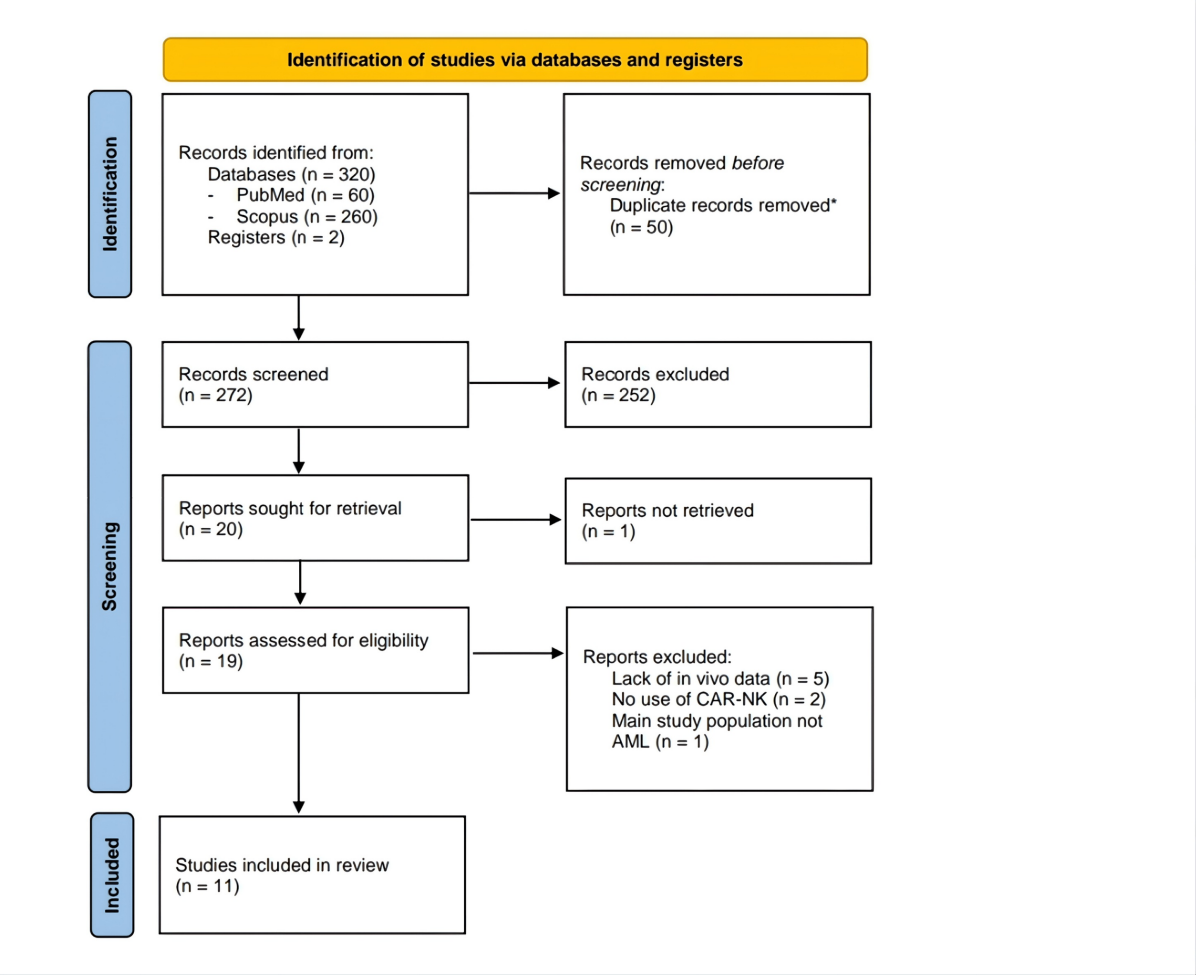
Article retrieval. Process following Preferred Reporting Items for Systematic Reviews and Meta-Analyses (PRISMA) guidelines [21]. * Duplicates were removed using the Rayyan platform [22].

Of 322 records identified, 19 were assessed in full text, and 11 met the inclusion criteria (Figure 1). Ten were preclinical mouse xenograft studies using human leukemic cells, two directly comparing CAR-NK with CAR-T [26,27], and one was a phase I clinical trial involving three patients (two adults, one pediatric) [28].

Most preclinical studies carried a high risk of bias, mainly from absent randomization, blinding, and sample size calculation, although all provided details on animal strain and transplantation methods (Table 1). By contrast, the only clinical study demonstrated good methodological quality, achieving a JBI score of 7 [28].

**Table 1:** Preclinical quality assessment using the Systematic Review Centre for Laboratory Animal Experimentation (SYRCLE) scale. Y: “YES,” low risk of bias; N: “NO,” high risk of bias; U: “UNCLEAR,” risk of bias cannot be adequately assessed [23].

Type of bias	Selection bias			Performance bias		Detection bias		Attrition bias	Reporting bias	Other
Domain	Sequence generation	Baseline characteristics	Allocation concealment	Random housing	Blinded interventions	Random outcome assessment	Blinded outcome	Incomplete outcome data	Selective reporting	Other sources of bias
Study	Outcome									
[29]	U	U	U	U	U	U	U	U	Y	Y
[30]	U	U	U	U	U	U	U	Y	Y	Y
[26]	U	U	U	U	U	U	U	Y	Y	Y
[31]	U	U	U	U	U	U	U	Y	Y	N
[27]	U	U	Y	U	U	U	Y	Y	Y	Y
[32]	U	U	Y	U	U	U	U	Y	Y	N
[33]	U	U	U	U	U	U	U	Y	Y	Y
[34]	U	U	U	U	U	U	U	Y	Y	N
[35]	U	U	Y	U	U	U	U	Y	Y	Y
[36]	U	U	Y	U	U	U	U	Y	Y	Y

Characteristics of Cell Models and In Vitro Cytotoxicity

All studies used allogeneic NK cells: 63% from peripheral blood and the remainder from the NK-92 line; only one study used umbilical cord blood (Table 2). All confirmed in vitro cytotoxicity against leukemic cells, with CD33 and CD123 being the most frequently evaluated antigens. All studies used IL-2 for CAR-NK expansion; several also employed feeder cells K562 [31-33] or closed-system expansion platforms (e.g., CliniMACS Prodigy®, Miltenyi Biotec, Bergisch Gladbach, Germany), achieving post-thaw viabilities >80% [30].

**Table 2:** General characteristics. NK: natural killer; CAR: chimeric antigen receptor; IL: interleukin; m: membrane-bound; s: secreted; hUC: human umbilical cord blood; N.R: not reported; PB: peripheral blood; *nanobody targeting CD16

Study	NK cell source	Antigen-binding domain	CAR generation	Costimulatory domain	Transduction method	Transduction efficiency
Albinger et al. 2022 [29]	PB	CD33	2nd	4-1BB-CD3ζ	Lentivirus	35-60%
Albinger et al. 2024 [30]	PB	CD33	2nd	4-1BB-CD3ζ	Lentivirus	28-32%
Caruso et al. 2022 [26]	PB	CD123	2nd	4-1BB-CD3ζ	Retrovirus	58,8%
Christodoulou et al. 2021 [31]	PB	CD123	2nd - 4th	2B4-CD3ζ	Retrovirus	21-98%
				4-1BB-CD3ζ		
				2B4-CD3ζ-sIL15		
Dong et al. 2022 [27]	PB	NPM1c + HLA-A2	2nd - 4th	4-1BB-CD3ζ	Lentivirus	65%
				4-1BB-CD3ζ-m15		
Du et al. 2021 [32]	PB	NKG2D	2nd - 4th	4-1BB-CD3ζ	Non-viral transposons	63-90%
				4-1BB-CD3ζ-sIL15		
Mansour et al. 2023 [33]	NK-92 - hUC	FLT3	2nd - 4th	CD28-CD3ζ	Lentivirus	46.30%
				2B4-CD3ζ		
				CD28-CD3ζ-sIL15		
Morgan et al. 2021 [34]	Non-irradiated NK92	CD123	4th	4-1BB-CD3ζ-hIL15	Retrovirus	N.R
Salman et al. 2019 [35]	NK-92	CD4	3rd	CD28-4-1BB-CD3ζ	Lentivirus	85%
Tang et al. 2018 [28]	NK-92	CD33	3rd	CD28-4-1BB-CD3ζ	Lentivirus	90%
Zhang et al. 2023 [36]	PB	CD33 + B16 VHH*	2nd	4-1BB-CD3ζ	Lentivirus	45-60%

Second-generation CARs incorporating 4-1BB or 2B4 domains showed superior cytotoxicity, while the addition of IL-15, whether secreted, membrane-bound, or complexed, enhanced persistence, activation, and effector function [27,31-34]. Cytokine-induced memory-like (CIML) CAR models showed increased cytotoxicity and proliferation [27].

Transduction was primarily viral, with reported efficiencies between 28% and 85% (Table 2). Notably, Du et al. [32] applied a non-viral PiggyBac transposon system, achieving 14% efficiency at day seven, 63% at day 28, and up to 90% by day 49 of culture.

In Vivo Effectiveness

Most studies used NSG mice, while only a few employed humanized models such as NSGS [29,30,34] or IL15-NOG [26]. Xenografts varied in cell type and dose, and only two studies tested primary AML blasts [33,34]. Therapy was administered primarily intravenously, with doses ranging from 1×10⁶ to 1×10⁷ in preclinical models and up to 5×10⁹ in the clinical trial [28].

Bioluminescence (BLI) analyses (Table 3) consistently showed reduced leukemic burden in CAR-NK groups compared to controls, particularly with NPM1c [27], 2B4.ζ [31], NKG2 [32], and CD4 [35] constructs. Nevertheless, progressive disease was observed in all groups, progressing faster without CAR therapy [26-36].

**Table 3:** Leukemic burden after treatment with CAR-NK cells. The values were digitized and estimated from figures, reported as mean ± SD when available/derivable; otherwise mean (range). NK: natural killer; CAR: chimeric antigen receptor; Ctrl: control; N.R: not reported; n.s: not significant; NT: no CAR transduction; PROD: Prodigy; SS: Small Scale; Tt: Time after treatment; (**) Only top bar reported; (†) Derived from SEM; (") p value taken from dot plot with individual points and mean or median, without error bars (reported as bar graph in the article).

Study	Xenograft (cells)	Type of transplanted cells	Leukemic burden				
		Treatment - Control	Tt	n	Total flux (p/s)	Statistical Test	p-value
					Mean ± SD / (range)		
Albinger et al. 2022 [29]	OCI-AML2	CD33-CAR-NK (1 dose)	21	7	1.2×10^8^ (1x10^8^ - 2x10^10^)	Median ± Range	N.R
		Ctrl: NT-NK			5×10^10^ (1.7x10^10^ - 2.4x10^9^)		
		CD33-CAR-NK (3 doses)	21	7	1.5×10^8^ (1x10^8^ - 4.8x10^8^)	Median ± Range	N.R
		Ctrl: NT-NK		6	1.8×10^10^ (1.4x10^10^ - 2.7x10^10^)		
Albinger et al. 2024 [30]	OCI-AML2	CD33-CAR-NK (PROD/ SS)	18	9	8.7x10^0^ (2.3x10^1^) **	Mean±SD	N.R
		Ctrl 1: CD33-CAR-NK		9	4.6x10^0^ (5.7x10^1^) **	Mean±SD	
		Ctrl: 2 NT-NK		7	1.1x10^6^ (8.2x10^8^) **	Mean±SD	
Caruso et al. 2022 [26]	THP-1	CD123-CAR-NK	50	4	5.4×10^5^ (4.8x10^5^ - 2.3x10^7^)	Median ± Range	N.R
		Ctrl 1: CD123-CAR-T			4.4×10^5 ^(3.2x10^5^ - 2.2x10^10^)		
		Ctrl 2: NT-NK	30		5.5×10^9 ^(2.3 - 6.6x10^9^)		
Christodoulou et al. 2021 [31]	MV-4-11	CD123-2B4.ζ CAR-NK	14	(8-12)	1.9×10^4^ (5.3x10^3^ - 9.4x10^4^)	ANOVA	<0.01
		Ctrl 1: 4-1BB.ζ CARNK			4.3×10^4^ (1.1x10^4^ - 9.5x10^4^)		
		Ctrl 2: NT-NK			1.2×10^5^ (2.8x10^4^ - 2.4x10^5^)		<0.0001
		2B4.ζ/sIL-15 CAR-NK	14	(5-7)	5.3×10^3 ^(2x10^3^ - 1.2x10^4^)	ANOVA	N.R
		Ctrl 1: 2B4.ζ CARNK			7.1×10^3 ^(1x10^3^ - 2.3x10^4^)		
		Ctrl 2: NT-NK			1.4×10^5^ (5.2x10^4^ - 2.9x10^5^)		<0.0001
	MOLM-13	2B4.ζ/sIL-15 CAR-NK	14	5	1.3×10^5 ^(1.6x10^4^ - 2.8x10^5^)	ANOVA	<0.0001
		Ctrl 1: 2B4.ζ CARNK			5.9×10^5 ^(4.3x10^5^ - 8.4x10^5^)		
		Ctrl 2: NT-NK			7.3×10^6 ^(4x10^6^ - 1.6x10^7^)		<0.0001
Dong et al. 2022 [27]	OCI-AML3	NPM1c-CAR-CIML NK	10	4	2 ± 0.6 x10^8 ^†	ANOVA	0.02
		Ctrl: NT-NK			4.3 ± 0.4 x10^8 ^†		
		NPM1c-CARm15-NK	18	4	5.3 ± 3.4 x10^8 ^†	ANOVA	0.05
		Ctrl 1: NPM1c-CAR-NK			11.2 ± 4.8 x10^8 ^†		
		Ctrl 2: NT-NK			14.6 ± 7.4 x10^8 ^†		0.05
		NPM1c-CARm15-NK	18	4	1.3 ± 1.2 x10^9 ^†	ANOVA	0.49
		Ctrl 1: NPM1c-CAR-T			1.2 ± 0.6 x10^9 ^†		
		Ctrl 2: NT-NK			2.8 ± 1.2 x10^9 ^†		0.001
Du et al. 2021 [32]	KG1	NKG2D CAR-NK	42	5	1.5×10^8 ^(1.4x10^8^ - 1.6x10^8^)	Median ± Range	N.R
		Ctrl: NT-NK			2.3×10^8 ^(1.9x10^8^ - 2.9x10^8^)		
		NKG2D CAR/IL15-NK (1 dose)	17	6	2.5×10^3 ^(1.3x10^3^ - 4.6x10^3^)	N.R	<0.05 "
		NKG2D CAR/IL15-NK (2 doses)		4	2.3×10^3 ^(1x10^3^ - 5.4x10^3^)		
		NKG2D CAR/IL15-NK (3 doses)		4	5.1×10^3 ^(2.6x10^3^ - 8.8x10^3^)		
		Ctrl 1: NKG2D CAR-NK		6	6.7×10^5 ^(4.5x10^4^ - 1.6x10^6^)		
		Ctrl 2: NT-NK		5	2.7×10^6 ^(2.4x10^6^ - 3.6x10^6^)		<0.0001 "
Mansour et al. 2023 [33]	MOLM-13	FLT3 CAR_CD28/CD3ζ	12	3	1.6 ± 0.52 x10^8 ^†	ANOVA	N.R
		Ctrl1: CAR 2B4/CD3ζ			2.3 ± 0.69 x10^8 ^†		
		Ctrl2: NT-NK-tCD19			5.3 ± 3.12 x10^8 ^†		<0.01
Morgan et al. 2021 [34]	LMA-PDX~	CD123-CAR-hIL15-NK-92 (1 dose)	14	7	39.2 ± 10 %†	Unpaired t-test	n.s
		Ctrl: NT-NK92		4	32.9 ± 7.4 %†		
		CD123-CAR-hIL15-NK-92 (2nd dose)	21	7	19.8 ± 12.4 %†	Unpaired t-test	0.05
		Ctrl: NT-NK92		4	61.9 ± 11.8 %†		
Salman et al. 2019 [35]	MOLM-13	CD4-CAR-NK	9	8	3.3 ± 2.8 x10^6^	N.R	< .00001
		Ctrl: NT-NK			0		

CAR-NK therapy significantly improved overall survival (OS), especially in CD123 [26], FLT3 [33], and bispecific CD33/B16 [36] models (Table 4). Only Christodoulou et al. [31] reported early toxicity and mortality, restricted to MV-4-11 xenografts treated with 2B4.ζ/sIL-15 CAR-NK.

**Table 4:** Overall survival analysis after treatment with CAR-NK cells. NK: natural killer; CAR: chimeric antigen receptor; Ctrl: control; FS: Final Survival; HSCs: haematopoietic stem cells; N.R: not reported; n.s: not significant; NT: no CAR transduction, T: Follow-up time; (**): Median not reached.

Study	Xenograft (cells)	Type of transplanted cells	Survival analysis				
		Treatment - Control	Statistical Test	T	Median (days)	n	p-value
Caruso et al. 2022 [26]	THP-1	CD123-CAR-NK	Mantel Cox	100	>80**	4	n.s
		Ctrl 1: CD123-CAR-T			60	4	
		Ctrl 2: NT-NK			40	4	0.02
	CD34+ HSCs	CD123-CAR-NK	Kaplan– Meier	15	>15**	6	N.R
		Ctrl 1: CD123-CAR-T			5	6	
		Ctrl 2: NT-NK			>15**	6	
Christodoulou et al. 2021 [31]	MV-4-11	CD123-2B4.ζ CAR-NK	Mantel Cox	80	63	(8-12)	<0.01
		Ctrl 1: 4-1BB.ζ CARNK			56	(8-12)	
		Ctrl 2: NT-NK			55	(8-12)	<0.05
		2B4.ζ/sIL-15 CAR-NK	Mantel Cox	80	26	(5-7)	<0.001
		Ctrl 1: 2B4.ζ CARNK			71	(5-7)	
		Ctrl 2: NT-NK			48	(5-7)	<0.001
	MOLM-13	2B4.ζ/sIL-15 CAR-NK	Mantel Cox	30	27	5	n.s
		Ctrl 1: 2B4.ζ CARNK			26	5	
		Ctrl 2: NT-NK			20	5	<0.01
Du et al. 2021 [32]	KG1	NKG2D CAR-NK	Log rank	55	52	5	<0.01
		Ctrl: NT-NK			45	5	
		NKG2D CAR/IL15-NK (1 dose)	Log rank	100	52	6	<0.0001
		NKG2D CAR/IL15-NK (2 doses)			67.5	4	
		NKG2D CAR/IL15-NK (3 doses)			>100**	4	
		Ctrl 1: NKG2D CAR-NK			44	6	
		Ctrl 2: NT-NK			41	5	
Mansour et al. 2023 [33]	MOLM-13	FLT3 CAR NK-92	Log rank	60	>60**	5	<0.01
		Ctrl: NT-NK			37		
		FLT3 CAR-sIL15 Off-the-shelf	Log rank	50	45	5	<0.05
		Ctrl1: sIL-15-NK			34		
		Ctrl2: NK-tCD19 (NT)			25		<0.001
	AML blast	FLT3 CAR-sIL15	Log rank	50	43	9	0.012
		Ctrl2: NT-NK			30		
Salman et al. 2019 [35]	MOLM-13	CD4-CAR-NK (2 doses)	Mantel Cox	20	20	8	0.0017
		Ctrl: NT-NK			15		
Zhang et al. 2023 [36]	THP1	CD33/B16 CAR-NK	Kaplan– Meier	60	>60**	4	N.R
		Ctrl 1: CD33 CAR-NK			37		
		Ctrl 2: NT-NK			27		

Combination strategies incorporating IL-15 into the CAR construct [27,31-34] generally prolonged survival and produced greater reductions in bioluminescence (Tables 3, 4). Du et al. [32] showed a clear dose-response: two and three infusions of NKG2D-CAR/IL-15 NK cells extended survival to ~60 and ~100 days, respectively, preventing the relapse observed after a single dose. Consistently, Morgan et al. [33] reported a significant further decrease in leukemic burden after a second dose of CD123-CAR/hIL-15 NK cells, an effect not achieved with a single infusion.

Two studies compared CAR-NK with CAR-T [26,27], finding no significant differences in leukemic burden or survival (Tables 3, 4).

Leukemic and NK Infiltration

Control groups exhibited substantially greater leukemic infiltration in bone marrow and spleen (~2-100%) than CAR-treated mice (~0-20%). An exception was Morgan et al. [34], who observed no reduction even after two doses of CD123-hIL15 CAR-NK (Table 5). The largest decreases were achieved with CAR-IL15 constructs [27,33], bispecific CD33/B16 CAR-NK [36], PROD-CAR-NK [30], and with multiple dosing regimens [29]. Dong et al. [27] additionally reported lower hepatic leukemic infiltration with NPM1c-targeted therapy compared to controls (not tabulated).

**Table 5:** Leukemic cell infiltration after treatment with CAR-NK cells. The values were digitized and estimated from figures, reported as mean ± SD when available/derivable; otherwise mean (range). NK: natural killer; CAR: chimeric antigen receptor; BM: bone marrow; Ctrl: control; L: liver; M: month; N.R: not reported; n.s: not significant; NT: no CAR transduction; PB: peripheral blood; PROD: Prodigy; SS: Small Scale; S: spleen; Tt: Time after treatment; (~) Cryopreserved AML cells from PB of a patient with secondary AML, FAB subtype M5 with ASXL1, FLT3, NF1, NPM1, RAD21, STAG1, TET2, and TP53 mutations; (†) Derived from SEM; (**) After chemotherapy, patients received three escalating doses of CAR-NK: Child (C) and Man (M) 3×10^8^ - 6×10^8^ - 1×10^9^; Woman (W) 1×10^9^ - 3×10^9^ - 5×10^9^.

Study	Xenograft (cells)	Type of transplanted cells	Leukemic burden							
		Treatment - Control	Tt	n	Bone Marrow	Statistical Test	p-value	Spleen	Statistical Test	p-value
					Mean ± SD / (range)			Mean ± SD / (range)		
Albinger et al. 2022 [29]	OCI-AML2	CD33-CAR-NK (1 dose)	21	7	0%	Median ± Range	N.R	0%	Median ± Range	N.R
		Ctrl: NT-NK			1.7 (1.4 -1.9%)			1.5 (0.5-2.7%)		
		CD33-CAR-NK (3 doses)	21	7	0%	Mann– Whitney	<0.0001	0%	Mann– Whitney	<0.0001
		Ctrl: NT-NK		6	9.3 (6.3 -12.1%)			5.6 (3.7 -7.6%)		
Albinger et al. 2024 [30]	OCI-AML2	CD33-CAR-NK (PROD y SS)	19	9	0.3 ± 0.2%	Student’s t test	0.23	0 ± 0.1%	Student’s t test	0.011
		Ctrl 1: CD33-CAR-NK		9	0.1 ± 0.2%			0%		
		Ctrl: NT-NK		7	2.9 ± 2%		0.0012	1.4 ± 0.7%		
Christodoulou et al. 2021 [31]	MV-4-11	CD123-2B4.ζ CAR-NK	22	(8-12)	58.7%	Mean	N.R	1.75%	Mean	N.R
		Ctrl: 4-1BB.ζ CARNK			74.1%			12.3%		
		Ctrl: NT-NK			90.8%			12.8%		
		2B4.ζ/sIL-15 CAR-NK	22	(5-7)	0.1%	Mean	N.R	3.2%	Mean	N.R
		Ctrl: 2B4.ζ CARNK			98.2%			97.7%		
		Ctrl: NT-NK			95%			98.1%		
Dong et al. 2022 [27]	OCI-AML3	NPM1c-CAR-CIML NK	10	4	6.8 ± 3.4 x10^5 ^†	ANOVA	0.06	1.4 ± 0.8 x10^4 ^†	ANOVA	0.06
		Ctrl: NT-NK			14.2 ± 3.6 x10^5 ^†			2.6 ± 0.6 x10^4 ^†		
		NPM1c-CARmb15-NK	18	4	5.3 ± 1 x10^6 ^†	ANOVA	0.03	1.1 ± 0.4 x10^5 ^†	ANOVA	0.03
		Ctrl: NPM1c-CAR-NK			7.6 ± 3.8 x10^6 ^†			2.1 ± 0.2 x10^5 ^†		
		Ctrl 2: NT-NK			13.6 ± 6.8 x10^6 ^†		0.2	2.7 ± 1 x10^6 ^†		0.34
Mansour et al. 2023 [33]	MOLM-13	FLT3 CAR-sIL15 Off-the-shelf	21	4	0%	ANOVA	n.s	N.R	N.R	N.R
		Ctrl1: sIL-15-NK			4.2 ± 1.6%					
		Ctrl2: NK-tCD19 (NT)			40 ± 13%		<0.01			
Morgan et al. 2021 [34]	LMA-PDX~	CD123-CAR-hIL15-NK-92 (2nd dose)	21	7	91.9 ± 6.35% †	Unpaired t-test	n.s	60.9 ± 14% †	Unpaired t-test	0.05
		Ctrl: NT-NK92		4	88.6 ± 9.8% †			88.5 ± 6.4% †		
Tang et al. 2018 [28]	C: LMA M4 ETO+, C-KIT+	Pre-treatment	1M / 4M	1**	4%	Mean	N.R	N.R	N.R	N.R
		Ctrl: Post-treatment			0 / 76%					
	M: LMA M4 t(3;16)	Pre-treatment	1M		27%					
		Ctrl: Post-treatment			75%					
	W: LMA M4	Pre-treatment	10		37.50%					
		Ctrl: Post-treatment			79.50%					
Zhang et al. 2023 [36]	THP1	CD33/B16 CAR-NK	21	4	7.7 ± 2.2%	Student’s t test	<0.01	N.R	N.R	N.R
		Ctrl 1: CD33 CAR-NK			39.3 ± 7.2%					
		Ctrl 2: NT-NK			95.7 ± 4.8%		<0.01			

NK infiltration was consistently higher in CAR-treated groups (Table 6), with ~0-99% positivity in bone marrow and spleen, versus ~0-24% (BM) and ~0-86% (spleen) in controls. Dong et al. [27] also noted greater hepatic infiltration with NPM1c CAR-NK therapy (not tabulated). The highest tissue levels were observed with CD123-2B4ζ CAR-NK ± IL-15 [31] and FLT3 CAR-sIL-15 [33]. Across models, NK presence declined over time [26-36], although repeat dosing improved tissue infiltration [29,34].

**Table 6:** Therapeutic cell infiltration after treatment with CAR-NK cells. The values were digitized and estimated from figures, reported as mean ± SD when available/derivable; otherwise mean (range). NK: natural killer; CAR: chimeric antigen receptor; BM: bone marrow; Ctrl: control; L: liver; M: month; N.R: not reported; n.s: not significant; NT: no CAR transduction; PB: peripheral blood; PROD: Prodigy; SS: Small Scale; S: spleen; Tt: Time after treatment; (~) Cryopreserved AML cells from PB of a patient with secondary AML, FAB subtype M5 with ASXL1, FLT3, NF1, NPM1, RAD21, STAG1, TET2, and TP53 mutations; (†) Derived from SEM; (**) After chemotherapy, patients received three escalating doses of CAR-NK: Child and Man 3×10^8^ - 6×10^8^ - 1×10^9^; Woman 1×10^9^ - 3×10^9^ - 5×10^9^.

Study	Xenograft (cells)	Type of transplanted cells	NK cell infiltration							
		Treatment - Control	Tr	n	Bone Marrow	Statistical test	p-value	Spleen	Statistical test	p-value
					Mean ± SD / (range)			Mean ± SD / (range)		
Albinger et al. 2022 [29]	OCI-AML2	CD33-CAR-NK (1 dose)	18	7	0.4 (0.3-0.5%)	Median ± Range	N.R	0.9 (0.5-1.6%)	Median ± Range	N.R
		Ctrl: NT-NK			0%			0%		
		CD33-CAR-NK (3 doses)	21	7	1.1 (0.7-1.5%)	Mann– Whitney	< 0.01	17.8 (9.8-26.1%)	Mann– Whitney	<0.001
		Ctrl: NT-NK		6	0.4 (0.3-0.5%)			2.2 (0.8-3.8%)		
Albinger et al. 2024 [30]	OCI-AML2	CD33-CAR-NK (PROD y SS)	19	9	0%	Mean± SD	N.R	0%	Mean± SD	N.R
		Ctrl 1: CD33-CAR-NK		9	0%			0%		
		Ctrl: NT-NK		7	0 ± 0.1%			0%		
Christodoulou et al. 2021 [31]	MV-4-11	CD123-2B4.ζ CAR-NK	22	(8-12)	39.8%	Mean	N.R	98%	Mean	N.R
		Ctrl: 4-1BB.ζ CARNK			24.2%			87%		
		Ctrl: NT-NK			8.4%			86%		
		2B4.ζ/sIL-15 CAR-NK	22	(5-7)	99%	Mean	N.R	99%	Mean	N.R
		Ctrl: 2B4.ζ CARNK			1.97%			2.39%		
		Ctrl: NT-NK			4.45%			1.9%		
Dong et al. 2022 [27]	OCI-AML3	NPM1c-CARmb15-NK	14	4	3.1 ± 1% †	ANOVA	0.03	1.1 ± 0.6% †	ANOVA	0.03
		Ctrl: NPM1c-CAR-NK			0.8 ± 0.2% †			0.1 ± 0.2% †		
		Ctrl 2: NT-NK			0%			0%		
Mansour et al. 2023 [33]	MOLM-13	FLT3 CAR-sIL15 Off-the-shelf	21	4	14.7 ± 2%	ANOVA	<0.01	N.R	N.R	N.R
		Ctrl1: sIL-15-NK			5.5 ± 2.9%					
		Ctrl2: NK-tCD19 (NT)			0%		<0.001			
Morgan et al. 2021 [34]	LMA-PDX~	CD123-CAR-hIL15-NK-92 (2nd dose)	21	7	0%	Unpaired t-test	n.s	28.8 ± 17.4% †	Unpaired t-test	0.05
		Ctrl: NT-NK92		4	0%			0%		
Zhang et al. 2023 [36]	THP1	CD33/B16 CAR-NK	21	4	1.7 ± 0.3%	Student’s t test	<0.05	N.R	N.R	N.R
		Ctrl 1: CD33 CAR-NK			0%					

In the clinical trial [28], three patients with relapsed/refractory AML (M4) received dose-escalated, irradiated CAR-NK-92 cells after chemotherapy. No objective responses were observed; all patients showed increasing blast counts, which led to treatment discontinuation (Table 5). The pediatric case had the latest relapse (four months) and showed transient molecular improvements (AML1/ETO: 1574→308; WT1: 247→12 per 10,000 ABL). Circulating CAR-NK-92 cells were detectable but short-lived, measuring 4×10² to 2.9×10³ cells/mL in the pediatric patient (days three to eight post-infusion) and 7.6×10² to 2.9×10³ cells/mL in the adult male over the same interval (not tabulated) [28].

Safety and Cytokine Profile

CAR-NK therapy induced increased activation and degranulation markers across models (IFN-γ, MIP-1α, GM-CSF, granulysin, granzymes A/B, sFasL) [26,29,30,34]. Caruso [26] additionally noted upregulation of CD16, CD57, and PD-1 in expanded NK cells, whereas Albinger [29] reported no significant differences from controls.

In preclinical studies, inflammatory cytokines (IL-6, IL-10, TNF-α) remained low [29,30,34]. In contrast, Tang et al. [28] observed transient increases of IL-6, IL-10, and IL-17A after CD33-CAR-NK-92 infusion in patients, resulting in grade I CRS that resolved within 24-48 hours without sequelae, and Morgan et al. [34] reported similar IL-10/IL-17A elevations in CD123-CAR-NK-92-treated mice without any complications. Christodoulou et al. [31] was the only study to describe increased hTNF-α with CD123-CAR-2B4ζ/sIL-15, accompanied by a sustained rise in hIL-15 (from <100 pg/ml at day 14 to >1000 pg/ml at day 28), correlating with early toxicity and mortality.

Safety was otherwise preserved. Mansour et al. [33] reported that FLT3 CAR-sIL-15 NK cells reduced tumor burden without affecting CD34⁺ HSC-derived populations in marrow, blood, liver, or spleen. Similarly, Caruso et al. [26] demonstrated a favorable profile for CD123-CAR-NK cells compared with CAR-T: CAR-T caused endothelial injury, marrow aplasia, and death, whereas CAR-NK maintained 100% survival without hematopoietic or endothelial toxicity.

Discussion

CAR-NK cells are attracting considerable interest as an emerging immunotherapeutic platform for acute myeloid leukemia. Preclinical studies in this review consistently show superior efficacy in eliminating leukemic cells compared to unmodified NKs, and a more proliferative and metabolically active phenotype (Tables 3-6). These properties position CAR-NK therapy as a potentially transformative approach within adoptive cell therapies; however, it is important to note that the high risk of bias reduces the overall strength and reliability of the evidence and should be carefully considered when interpreting these results.

Similarly, clinical translation remains at a very early stage. The single-phase I trial failed to show meaningful efficacy, reflecting the intrinsic limitations of CAR-NK cells, and especially the restricted activity of irradiated NK-92 cells [28,37,38]. This combination of limited clinical data and the high risk of bias in preclinical evidence underscores a central challenge for the field: how to convert promising laboratory advances into durable therapeutic benefit for patients. 

Manufacturing and engineering hurdles persist. Gene transfer still relies predominantly on viral vectors, with lentiviral transduction being the most used approach in this review (Table 2); which, while effective, raises concerns about insertional mutagenesis and imposes costly, labor-intensive processes [39-41]. Non-viral platforms (e.g., PiggyBac, Sleeping Beauty) offer scalable, lower-cost alternatives and are beginning to show workable efficiencies in NK engineering, but require further safety validation [32,42,43].

Persistence is equally critical. Several strategies have been explored to prolong CAR-NK survival and function, including cytokine supplementation, feeder cell-based expansion, and the incorporation of constitutive IL-15 into CAR designs. Results, however, are mixed: IL-2, long used to support NK cell growth, is limited by adverse effects (cytokine storm, endothelial cell damage) and T-reg activation [44-47]. IL-15 more effectively sustains proliferation and cytotoxicity, yet its short half-life necessitates continued dosing, raising concerns about systemic toxicity and NK cell exhaustion [47-49]. Meanwhile, modified K562 feeder cells support NK expansion and reduce post-infusion dysfunction, but their malignant origin and the associated risk of contamination demand rigorous (and costly) release testing to ensure clinical safety [50,51].

Therefore, more innovative strategies, including constitutive IL-15 CARs and preactivation with IL-12/15/18 (CIML), are emerging as a new approach given their improved persistence and greater antitumor activity in several models [52-55]. Collectively, studies incorporating combined expansion/persistence strategies demonstrated enhanced cytotoxicity, persistence, and survival benefits in this review (Tables 3-6). Nonetheless, progressive leukemic burden remained a recurring limitation even with CAR-based interventions [26-36], underscoring the need for designs that decouple durability from toxicity and further optimize the activity in disease sites.

Tissue infiltration further complicates the picture, with consistently low persistence of CAR-NK cells in bone marrow and spleen [26-36]. While restricting long-term persistence has been a safety goal, particularly with CAR-T to avoid myeloablation, emerging evidence suggests marrow NK proportions may carry prognostic value in leukemia [56]. Trafficking cues, such as CXCR4 overexpression, represent rational pathways to improve marrow homing and bolster antileukemic effects [57].

Overall, security was favorable in most of the studies analyzed. While the clinical trial reported only mild, self-limited adverse events [28], Christodoulou et al. [31] described significant toxicity and early mortality in mice treated with CD123-sIL15 CAR-NK; although CRS was unlikely due to the absence of murine inflammatory cytokines, this pattern raises concern for alloreactivity or on-target/off-tumor effects, given CD123 expression on endothelial and hematopoietic cells [31,58].

In my view, these adverse outcomes likely reflected a convergence of target selection on healthy tissues, feeder-driven hyperproliferation, and supra-physiologic IL-15 signaling. This interpretation is reinforced by other studies reporting no toxicity when constitutive IL-15 or feeder support is paired with alternative antigens (NKG2D, FLT3) [32,33], and satisfactory tolerance of CD123-CARs without constitutive IL-15 or feeder dependence [26,34].

Despite these obstacles, CAR-NK cells offer distinct advantages: a favorable safety profile with low rates of CRS and no reported neurotoxicity [26-36], CAR-dependent and CAR-independent cytotoxicity [15], a robust cytokine and cytotoxic protein secretion that broaden malignant-cell recognition [26,29,30,34], multiple sources of NK (peripheral blood, umbilical cord blood, or iPSCs), and lower manufacturing cost; enabling standardized, “off-the-shelf” products that improve access and scalability [26,30,33].

Looking ahead, progress will require innovation and smarter solutions that strengthen trafficking and marrow infiltration, improve persistence without undue toxicity, and prevent on-target/off-tumor damage. Emerging tools such as nanobody-based CARs, CRISPR/Cas9 genome editing, bispecific or adapter CARs, and inducible suicide genes represent promising next steps [36,59-62]. But technology alone won’t be enough; impact will also depend on stronger study designs, standardized models, and larger patient cohorts. Only through this combination can CAR-NK therapy move from a promising concept to a reliable therapy in oncology.

As mentioned before, this review has limitations. The risk of bias demonstrated unclear performance in several SYRCLE domains (Table 1), with substantial methodological issues, small sample sizes, and heterogeneity in experimental models, doses, and administration methods; on the clinical side, the available evidence is further constrained by the very limited number of patients studied, which restricts interpretability and weakens the strength of the conclusions.

## Conclusions

CAR-NK cells are becoming a promising therapeutic strategy, with features that make them particularly attractive for patients with AML. They may offer a flexible and complementary approach that expands available treatment options; however, the long-term benefits in this regard will depend on addressing key challenges in production and persistence to ensure that initial progress can lead to reproducible and meaningful clinical outcomes. Sustained progress in CAR-NK cell design and manufacturing could help establish this therapy as a viable option for AML, ultimately supporting more personalized care.

## Appendices

PubMed

((("leukemia, myeloid, acute"[MeSH Terms] OR ("leukemia"[All Fields] AND "myeloid"[All Fields] AND "acute"[All Fields]) OR "acute myeloid leukemia"[All Fields] OR ("acute"[All Fields] AND "myeloid"[All Fields] AND "leukemia"[All Fields])) AND ("immunotherapy"[MeSH Terms] OR "immunotherapy"[All Fields] OR "immunotherapies"[All Fields] OR "immunotherapy s"[All Fields]) AND ("killer cells, natural"[MeSH Terms] OR ("killer"[All Fields] AND "cells"[All Fields] AND "natural"[All Fields]) OR "natural killer cells"[All Fields] OR ("nk"[All Fields] AND "cells"[All Fields]) OR "nk cells"[All Fields]) AND ("receptors, chimeric antigen"[MeSH Terms] OR ("receptors"[All Fields] AND "chimeric"[All Fields] AND "antigen"[All Fields]) OR "chimeric antigen receptors"[All Fields] OR ("chimeric"[All Fields] AND "antigen"[All Fields] AND "receptor"[All Fields]) OR "chimeric antigen receptor"[All Fields]) AND "loattrfree full text"[Filter]) Filters: English, Spanish

Scopus

TITLE-ABS-KEY(leukemia, myeloid, acute AND immunotherapy AND killer cells, natural AND receptors, chimeric antigen) AND ( LIMIT-TO ( OA,"all" ) ) AND ( LIMIT-TO ( LANGUAGE,"English, Spanish" ) )
